# Invisible but Insidious Effects of Microplastics

**DOI:** 10.3390/molecules29235776

**Published:** 2024-12-06

**Authors:** Natalia A. Stefanova, Yulia S. Sotnikova, Aleksandra E. Osechkova, Elena V. Karpova, Dmitriy N. Polovyanenko, Anzhella Zh. Fursova, Daria A. Kiseleva, Tatyana G. Tolstikova, Nataliya G. Kolosova, Elena G. Bagryanskaya

**Affiliations:** 1 Institute of Cytology and Genetics, Siberian Branch of the Russian Academy of Sciences, Lavrentjev Avenue 10, 630090 Novosibirsk, Russia; anzhellafursova@yandex.ru (A.Z.F.); kolosova@bionet.nsc.ru (N.G.K.); 2 Department of Natural Sciences, Novosibirsk State University, 1 Pirogova str., 630090 Novosibirsk, Russia; 3 N.N. Vorozhtsov Novosibirsk Institute of Organic Chemistry, Siberian Branch of Russian Academy of Sciences, Lavrentjev Avenue 9, 630090 Novosibirsk, Russia; sotnikova@nioch.nsc.ru (Y.S.S.); osechkova@nioch.nsc.ru (A.E.O.); karpovae@nioch.nsc.ru (E.V.K.); dpolo@nioch.nsc.ru (D.N.P.); dasha.halikova@mail.ru (D.A.K.); tolstiktg@nioch.nsc.ru (T.G.T.); 4 State Novosibirsk Regional Clinical Hospital, St. Nemirovich-Danchenko 130, 630087 Novosibirsk, Russia; 5 Department of Ophthalmology, Novosibirsk State Medical University, Pr. Krasny 52, 630091 Novosibirsk, Russia

**Keywords:** microplastic, polymers, memory, cognitive ability, Alzheimer’s disease, age-related macular degeneration, OXYS rats

## Abstract

Increasing evidence on the adverse health impacts of microplastics (MPs) is available, but their associated risks to the well-being of humans and long-term impacts are poorly understood. An indicator of the remote effects of MPs may be their influence on the rate of aging. To assess the effects of MPs on the aging process, we used accelerated senescence OXYS rats that develop a complex of geriatric diseases. We prepared the polyethylene terephthalate MPs (2–6 microns in size) and in OXYS and Wistar (maternal strain) rats assessed the influence of chronic administration of MPs (10 or 100 mg/kg per day from age 1.5 to 3.5 months,) on the hematological and biochemical blood parameters, spatial learning, and memory. In addition, the effects of MPs on the development of cataracts and retinopathy, similar to age-related macular degeneration (AMD), in OXYS rats were assessed. We found that in the absence of significant changes in standard clinical blood parameters, chronic MP administration negatively affected the cognitive functions of both Wistar rats and OXYS rats. Additionally, a dose of 100 mg/kg MPs contributed to cataract and AMD progression in OXYS rats. Our results suggest that MPs may increase the rate of aging and, in the long term, lifespan.

## 1. Introduction

Widespread use, increased production, and inadequate disposal of plastic have led to plastic pollution, which has emerged as one of the most challenging contaminants worldwide. Approximately 10% of the total plastics produced annually accumulate as debris in the aquatic environment [1]. As environmental plastic waste degrades, it creates an abundance of diverse microplastic particles (diameter 0.1 µm to 5 mm) and secondary nanoplastics (diameter < 100 nm). Nano/microplastics have been detected in various regions of the human body, including the blood, organs, placenta, breast milk, and gastrointestinal system [2]. Microplastics (MPs) have long been considered inert [3]. However, there is growing evidence that MP exposure in humans may result in damage to the reproductive system, nervous system, cardiovascular system, respiratory system, digestive system, immune system, endocrine system, and motor system [4,5,6,7,8,9,10,11,12,13]. Compelling evidence in experimental animals has demonstrated that MP administration leads to various aspects of dysfunction in peripheral tissues/organs as well as in the brain: PS-MP can cross the blood–brain barrier, affect synaptic plasticity, induce neuroinflammation, and impair learning and memory in the hippocampus [14].

Recently, the World Health Organization recognized its negative effects on humans, which has sparked increased interest in this line of research. However, despite numerous statements about the toxic effects of MPs, there is currently no evidence of their direct harmful effects on animal and human health at environmentally (ecologically significant) concentrations [15]. It can be expected that MPs can cause harm to health with chronic exposure, but it is impossible to isolate their effects against the background of the cumulative effects of many stressors affecting the human body. The optimal approach for studying the possible mechanisms of potential toxicity through which MPs may affect human health is the use of animal models [16]. However, even in this case, a question arises about the choice of parameters, the state of which should be monitored for an objective assessment of the cumulative effects and remote consequences of exposure to MPs. An objective indicator of the impact of a particular factor on health can be its effect on the rate of aging, which manifests as the development of age-related diseases. The first study comparing the effects of MPs on young and old animals was recently published [17]. Its authors demonstrated greater vulnerability to the negative effects of three-month exposure to MPs in 18-month-old mice compared to 3-month-old mice. At the same time, there is no information on the effect of MP exposure on the aging process and the development of associated diseases, which in real life begins already in infancy.

In this study, we assessed the effects of chronic MP administration on the health of Wistar and OXYS rats, a unique model of accelerated senescence, which manifests as the early development of a complex of geriatric diseases, including cataracts and retinopathy, which corresponds to age-related macular degeneration (AMD) in humans and accelerated brain aging with signs of Alzheimer’s disease (AD) [18,19]. Polyethylene terephthalate (PET) was chosen as the model plastic. The PET industry capacity was 36.23 million tonnes per annum (mtpa) in 2023 and is expected to increase by more than 3% from 2023 to 2028. In addition, the increasing consumption of PET in the packaging, food and beverage, and textile industries makes PET one of the major environmental pollutants among all plastics used. In this context, the use of PET as a model plastic is more rational than the use of polystyrene spheres, which are widely used in similar studies [20]. We prepared the PET MPs (2–6 microns in size) from a drinking water bottle. Animals were fed MPs from the age of 1.5 to 3.5 months, during the period of active manifestation of cataracts, retinopathy, and signs of accelerated brain aging in OXYS rats. We assessed the impact of MPs on the hematological and biochemical profiles of OXYS and Wistar rats and their spatial learning and memory in the Barnes maze task. In addition, the effects of MPs on the development of cataracts and retinopathy, similar to AMD, in OXYS rats were assessed.

## 2. Results

### 2.1. PET Particle Preparation and Characterization

The synthesis of PET microparticles was accomplished by fully diluting the PET material in TFA and then controlling the precipitation by adding the PET solution to the water-enriched TFA solution, resulting in a mixture of microsized PET particles. Microparticles exceeding a diameter of 26 μm were isolated by filtration of the resulting suspension through a stainless-steel filter mesh with a mesh size of 26 μm.

**Figure 1:**
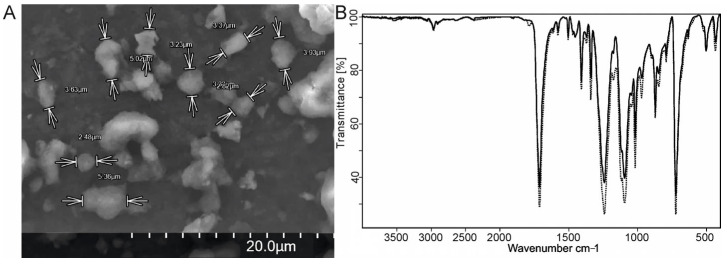
Characterization of the obtained particles: (**A**) SEM image; (**B**) FTIR spectra of the PET particles: initial film (solid line) and microparticles (dotted line).

The PET microparticles were characterized via SEM (Figure 1A). The study of several samples of prepared particles revealed a particle size range of 2–6 μm. An analysis of the authentic PET film and the PET microparticles conducted via the FTIR method revealed that the structure and chemical composition of the PET remained unaltered (Figure 1B).

### 2.2. Body Weight

MP exposure did not affect the body weights of either Wistar or OXYS rats (F_2,84_ = 1.74, *p* = 0.18), which were lower in OXYS rats (F_1,84_ = 5.12, *p* < 0.0001). In addition, MPs did not affect the relative weights of the brain, liver, heart, or testicles of either Wistar or OXYS rats. MPs affected only the kidney index (F_2,54_ = 5.18, *p* < 0.009), but as the analysis within the strains showed, the effect was significant only in OXYS rats (F_2,27_ = 7.95, *p* < 0.0019) against the background of both doses of MP exposure; this indicator decreased in a dose-dependent manner—by 6 and 8%, respectively.

### 2.3. Ophthalmoscopic Examination

**Figure 2:**
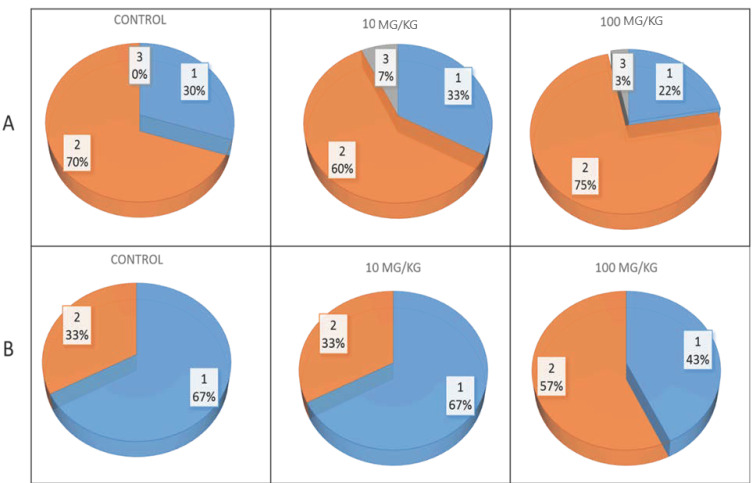
Effect of MPs on cataract (**A**) and retinopathy (**B**) development in OXYS rats. The data are presented as the distribution of the eyes of the animals within the cataract stages and signs of AMD-like pathology development in the control and MP-exposed OXYS rats. 1–3—corresponding stage of the disease.

Before the start of the behavioral tests, an ophthalmologist examined the OXYS and Wistar rats at the age of 3.5 months. During inspection, we did not find a single eye that was free of pathological changes in the lens or retina. MP exposure had no significant effect on the development of pathological changes in the lenses of OXYS rats, but against the background of plastic intake at a dose of 100 mg/kg, the percentage of eyes with more pronounced stages of cataracts (2nd and 3rd) slightly increased (Figure 2A). Additionally, a dose of 100 mg/kg of MPs contributed to AMD progression: MPs increased the proportion of eyes with the second, more pronounced stage of the disease by 1.7 times (Figure 2B). Neither control nor MP-exposed Wistar rats presented pathological changes in the retina or lens.

### 2.4. Behavior and Cognitive Ability in the Barnes Maze

**Figure 3:**
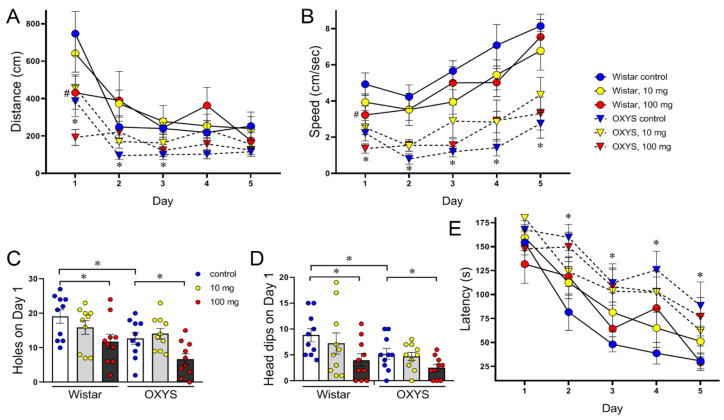
Barnes maze performance of MP-exposed and control OXYS and Wistar rats. Analysis of (**A**) the distance travelled by the rats to locate the escape box and (**B**) their speed. OXYS rats travelled a shorter distance to escape and at a slower speed than did Wistar rats. On day 1, the distance travelled and speed of MP-exposed Wistar rats at a dose of 100 mg/kg were significantly lower than those of control Wistar rats. The numbers of (**C**) holes and (**D**) heads dipping from the table on the first day of Barnes performance before training. The control OXYS rats and MP-exposed rats of both strains presented lower exploratory activity than the control Wistar rats did (*, Newman–Keuls post hoc test; *p* < 0.05). (**E**) The primary latency was longer in OXYS rats than in Wistar rats. The data are presented as the means ± SEMs, n = 9–10. In (**A**,**B**,**E**), * *p* < 0.05 for differences between control OXYS and Wistar rats; # *p* < 0.05 for an effect of MP exposure. The circles in (**C**,**D**) indicate the individual scores of each rat.

In habituation, when the animals were naive and untrained, the distance and speed were lower in OXYS rats (F_1,54_ = 13.53, *p* < 0.001 and F_1,54_ = 28.34, *p* < 0.0001, respectively) and in MP-exposed rats of both strains (F_2,54_ = 5.36, *p* < 0.01 and F_2,54_ = 4.21, *p* < 0.05, respectively; Figure 3A,B). On the first day, the numbers of holes and head dips from the table reflect the exploratory activity and anxiety level of the animals: more anxious rats explore fewer objects than do less anxious rats. All these parameters were lower in OXYS rats (F_1,54_ = 8.68, *p* < 0.005 and F_1,54_ = 6.02, *p* < 0.02, accordantly) and in MP-exposed rats of both strains (F_2,54_ = 7.66, *p* < 0.002 and F_2,54_ = 4.68, *p* < 0.02, accordantly; Figure 3C,D), thus reflecting decreased locomotion and exploratory activity and increased anxiety.

Over the 4 days of training, both Wistar and OXYS rats learned the location of the escape box. Our analysis revealed that the distance travelled to the target and the speed changed as the animals learned the position of the escape box (Figure 3A,B) and remained lower in OXYS rats (F_1,53_ = 17.94, *p* < 0.0001 and F_1,53_ = 46.15, *p* < 0.0001, respectively). However, the primary latency was longer in OXYS rats across days (F_1,54_ = 16.01, *p* < 0.001; Figure 3E). There were no overall effects of MPs on the distance measure (F_2,53_ = 1.84, *p* = 0.17), speed measure (F_2,53_ = 0.02, *p* = 0.98), or primary latency (F_2,54_ = 0.13, *p* = 0.88).

**Figure 4:**
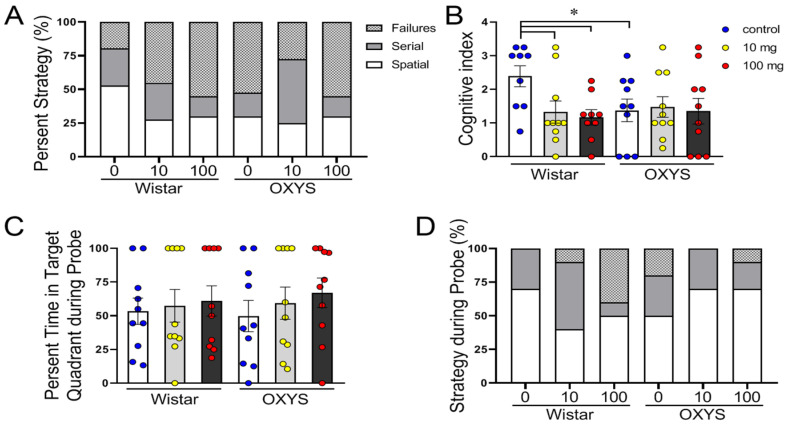
Cognitive performance of MP-exposed and control OXYS and Wistar rats in the Barnes maze. (**A**) Representative between-group comparison of profiles of strategy selection by each group for the entire training session (prior to probe). (**B**) A comparison of the summed cognitive indices for the training trials revealed that control OXYS rats and MP-exposed rats of both strains had significantly lower scores than control Wistar rats did (*, Newman–Keuls post hoc test; *p* < 0.05). (**C**) Percentage of time spent in the target quadrant during the probe trial. (**D**) Profiles of strategy selection by each group for the probe trial. The data are presented as the means ± SEMs; n = 9–10. In (**B**), * *p* < 0.05 for differences between control OXYS and Wistar rats and for an effect of MP exposure in Wistar rats. The circles in (**B**,**C**) indicate the individual scores of each rat.

Hippocampus-dependent changes in performance were observed in OXYS rats and in MP-exposed Wistar rats at both doses (Figure 4A). The control OXYS rats, as well as the MP-exposed OXYS and Wistar rats, preferred nonspatial (failures or serial) strategies (approximately 75% of the time) to spatial strategies (approximately 25%) over their control Wistar rats (approximately 50%) (F_2,159_ = 5.78, *p* < 0.005).

The cognitive scores for each animal across all training days were summed, and the mean cognitive index scores for each group were compared using one-way ANOVA (Figure 4B). Animals that used spatial strategies had higher cognitive index scores compared to those using the serial and failures strategies. Compared with the control Wistar rats, only the control Wistar rats had significantly greater cognitive indices (*p* < 0.05).

In the probe trial on day 7, we did not observe any difference in the percentage of time spent in the target quadrant (*p* > 0.05; Figure 4C), and more often, the rats used the spatial strategy (F_2,162_ = 16.25, *p* < 0.0001; Figure 4D). Notably, in Wistar rats, 40% of the MP-exposed animals failed at a dose of 100 mg/kg, whereas none of the control animals failed.

### 2.5. Hematologic and Biochemical Indicators in the Blood

Hematologic and biochemical blood parameters are normally used to evaluate the health of humans and animals. MP exposure did not affect hematological parameters, such as white blood cells (WBCs) or WBC subtypes, red blood cells (RBCs), hemoglobin (HGB), hematocrit (HCT), platelets, mean corpuscular volume (MCV), mean corpuscular hemoglobin (MCH), or mean corpuscular hemoglobin concentration (MCHC), in either Wistar or OXYS rats (Supplementary Table S1).

We examined essential biochemical parameters for assessing kidney and liver function, cardiovascular health, and bone metabolism. Two-way ANOVA revealed that the level of LDL depends on the genotype of the animals; i.e., the level of LDL was lower in OXYS rats (F_1,39_ = 58.4, *p* < 0.0001), and the intake of MP affected it: F_2,39_ = 4.8, *p* < 0.013. However, pairwise comparisons revealed that in Wistar rats, MPs at a dose of 100 mg/kg slightly but significantly reduced LDL levels, whereas in OXYS rats, MPs at a dose of 10 mg/kg increased LDL levels (by 10%). The HDL level was dependent on genotype and was greater in OXYS rats (F_1,39_ = 7.8, *p* < 0.01), and MPs did not significantly affect HDL levels (F_2,39_ = 3.00, *p* = 0.06). According to factor analysis, the ALT level did not depend on the genotype (F_1,39_ = 1.11, *p* = 0.29), but it was affected by the intake of plastic: F_2,39_ = 4.59, *p* = 0.016. However, pairwise comparisons revealed reliable differences only between the groups of OXYS rats exposed to MPs at doses of 10 and 100 mg/kg. Genotype influenced the AST level at the tendency level (F_1,39_ = 4.08, *p* = 0.050), whereas plastic intake did not affect this indicator (F_2,39_ = 0.11, *p* = 0.90).

The creatine kinase level also did not differ between Wistar and OXYS rats (F_1,39_ = 0.05, *p* = 0.90), but a two-factor ANOVA revealed the effect of MP exposure on creatine kinase activity (F_2,39_ = 3.40, *p* < 0.044), with a slight increase (*p* < 0.034) against the background of MP exposure at a dose of 10 mg/kg. Moreover, the effect was not significant in pairwise group comparisons. MPs did not affect the level of alkaline phosphatase (ALKP) (F_2,39_ = 0.82, *p* = 0.45), which was reduced in OXYS rats (F_1,39_ = 10.98, *p* < 0.002) and is associated with early osteoporosis development, which is one of the manifestations of accelerated senescence [18]. MPs changed only one biochemical parameter. The bilirubin level did not depend on the genotype of the animals (F_1,37_ = 0.81, *p* = 0.37), but it was affected by the intake of MPs (F_2,37_ = 4.80, *p* < 0.014): at a dose of 10 mg/kg, the bilirubin level significantly increased in both Wistar rats (*p* < 0.003) and OXYS rats (*p* < 0.021) to 26.5% and 39%, respectively (Supplementary Table S2).

## 3. Discussion

While there is already a considerable and robust body of evidence on the adverse health impacts of MPs, their associated risks to the well-being of humans and ecosystems have yet to be fully established, often overlooking their long-term impacts [21,22]. Moreover, some researchers have even argued that exposure to microplastics does not pose a risk due to their low concentration in the environment [23,24]. We believe that an indicator of the remote effects of MPs may be their influence on the rate of aging and, in the long term, on life expectancy. Age-related diseases are a natural manifestation of aging. Their development at an earlier age is considered a manifestation of premature aging, and at a later age, they become the basis for successful aging and longevity. More than 60% of people over 65 years of age suffer from not one but several diseases, and the “set” and age of manifestation are determined by genetic and environmental factors and quality of life. In this study, to assess the effects of MPs on the aging process, we used accelerated senescence OXYS rats, which at 3 months of age already develop a complex of geriatric diseases, including cataracts, AMD-like pathology, and the first signs of AD-like pathology [7]. Therefore, to assess the influence of MPs on the aging process, the period of active manifestation of these signs was chosen: from 1.5 to 3.5 months.

It has been shown [25] that the toxic effects of MPs on animal models depend on the size, polymer type, concentration, and exposure time of the MPs, which could significantly affect the biological endpoints. We evaluated the effects of several different concentrations of MPs (PET, one of the major environmental pollutants among all plastics) on hematologic and biochemical blood parameters, which are normally used to evaluate the health of humans and animals. Doses of 10 and 100 mg/kg were selected in this study, which corresponded to or was 10-times greater than the human exposure dose [26,27]. No effects of MPs on most of these parameters were found in either OXYS or healthy Wistar rats. The exception was that at a dose of 10 mg/kg, MPs significantly increased the bilirubin level in both Wistar and OXYS rats. Increased bilirubin levels may be caused by impaired liver function and/or increased breakdown of red blood cells. However, we did not detect changes in the levels of ALT and AST enzymes, which reflect liver dysfunction, or any changes in red blood parameters. In the absence of changes in most standard clinical health indicators, chronic MP intake negatively affected the behavior of OXYS rats and contributed to the progression of accelerated manifestations of brain aging. In addition, MP intake contributed to the progression of AMD-like pathology, increasing the severity of pathological changes in the retina. The effect of MPs on the development of cataracts was not as convincing, but it can be expected that with prolonged intake, the effects of MPs would be more pronounced.

Early cataract development is a basic trait for the selection of OXYS rats and results in the development of accelerated aging syndrome. The first signs of crystalline lens opacification are visible in 20–25% of OXYS rats to the age of 1.5 months, and by the age of 3 months, the incidence of the disease reaches 100% [18,28]. In the present study, ophthalmoscopic examination revealed signs of cataracts in all eyes of the 3.5-month-old control and MP-exposed OXYS rats, whereas no significant differences in the severity of pathological changes in the lenses were found. Moreover, the percentage of eyes with more pronounced stage 2 and 3 cataracts in those treated with MPs at a dose of 100 mg/kg was slightly greater than that in the control (78% and 70%, respectively).

In addition to cataracts, OXYS rats develop retinopathy similar to AMD in humans. AMD is a multifactorial neurodegenerative disease of the retina and is the main cause of vision deterioration and loss in individuals aged 60 years and older in developed countries [29]. In OXYS rats, the clinical manifestations of retinopathy develop by the age of ~3–4 months in the background of structural and functional changes in retinal pigment epithelium cells and disruption of choroidal microcirculation. These changes progress with age and lead to the death of photoreceptors, which is the basis of vision loss in AMD [18,28]. During ophthalmoscopic examination, pathological changes in the fundus were detected in the eyes of all control and MP-exposed OXYS rats. At a dose of 100 mg/kg, MPs promoted retinopathy progression, increasing the proportion of eyes with signs of greater severity in the second stage of AMD from 33 to 57%.

In parallel with early neurodegenerative changes in the retina in OXYS rats, neurodegenerative processes spontaneously occur in the brain—all key pathogenetic and “clinical” signs of AD develop [19]. The sequence of these diseases is as follows: dysfunction of mitochondria, hyperphosphorylation of tau protein, disruption of long-term post-tetanic potentiation, synaptic insufficiency, destructive changes in neurons, behavioral disorders and a decrease in cognitive functions in the early stages and their progression against the background of an increase in the level of APP, and increased accumulation of Aβ and the formation of amyloid plaques in the brain, corresponding to modern ideas about the pathogenesis of the sporadic form of this disease in humans [19]. Here, we demonstrated that MP has a negative effect on the behavior and cognitive abilities of rats. The effects of MPs depend on genotype, and it was surprising that the effects of MPs on the cognitive functions of healthy Wistar rats were more pronounced. The long-term effects of MPs may be unclear, but it can be assumed that chronic intake of MPs contributes to accelerated brain aging and the development of Alzheimer’s-type dementia. Indeed, MPs may specifically influence the folding of proteins, induce the formation of aberrant amyloid proteins, and, therefore, potentially trigger the development of systemic and local amyloidosis [30]. In APP/PS1 double-transgenic AD mice, intravenous administration of polystyrene MPs promoted the progression of cognitive impairment by inducing neuroinflammation [31]. In addition, nanoplastics increased amyloid-β peptide aggregation in vitro [30] and memory impairment and microglial activation [32]. However, the results of these studies might lead to the misestimation of the health risks of MP exposure to mammals because they used gavage experiments with MPs. Therefore, we conducted experiments on rats on the basis of the type and concentration of MPs to which humans are exposed in their daily life. MPs decreased locomotor and exploratory activity and increased anxiety in both Wistar and OXYS rats. Importantly, during the learning phase, all groups of rats, excluding the control Wistar rats, more often used nonhippocampal strategies, such as serial and failure searches.

## 4. Materials and Methods

### 4.1. Ethics Statement

The keeping of animals (including appropriate facilities, qualified personnel, and necessary documentation) and all experiments with animals were conducted according to Directive 2010/63/EU of the European Parliament and of the European Council of 22 September 2010 and was approved by the Commission on Bioethics at the ICG SB RAS (decision # 34 of 15 June 2016), Novosibirsk, Russia.

### 4.2. PET Particle Preparation and Characterization

**Figure 5:**
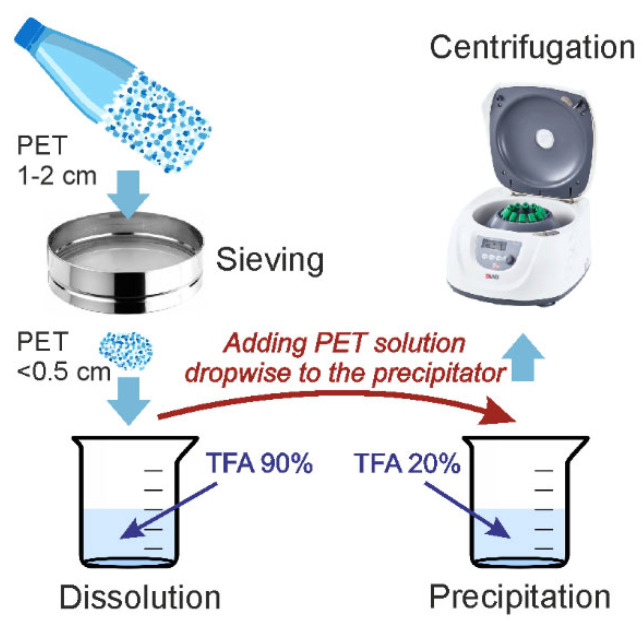
Schematic description of the method used to produce PET microparticles.

In this study, we adapted the approach to the synthesis of PET particles, as described by a group of scientists in a published paper, to align with our research objectives [33]. The PET used to produce the microparticles was obtained from a drinking water bottle. The methodology employed in the production of PET microparticles is illustrated in Figure 5. The bottle was cut into small fragments of approximately 1–2 cm. The particles were then ground in an IKA M20 mill (IKA Werke, Staufen im Breisgau, Germany), with the PET particles precooled with liquid nitrogen. This material was then fractionated via a 0.5 cm mesh sieve. Particles smaller than 0.5 cm were used for further experiments. Five grams of PET particles were dissolved in 180 mL of concentrated trifluoroacetic acid (TFA, 99%, Panreac, Barcelona, Spain) solution (90% *v*/*v*) under stirring at 70 °C and stirred until complete dissolution (4 h). Following dissolution of the solution, it was left to stand overnight. To precipitate the microparticles, the resulting PET solution was added dropwise to 180 mL of dilute aqueous trifluoroacetic acid (20% *v*/*v*) under stirring and heated to 70 °C for 4 h and then left overnight. Following the sedimentation of the particles, the supernatant was removed. Distilled water was then added, and the suspension was stirred. The suspension was subjected to centrifugation at 5000× *g* for 10 min, after which the supernatant was discarded. This process of adding a clean portion of water and centrifugation was repeated five times to remove residual TFA. Thereafter, the particles were transferred to ethanol for characterization studies. The particles were subsequently subjected to a drying process.

SEM–EDS analysis of the obtained MP particles was performed via an SU1000 FlexSEM II scanning electron microscope (Hitachi, Tokyo, Japan). A sample (10 μL) of MP suspension in ethanol with CaCO_3_ for particle dispersion was placed onto conductive tape on the stage and coated with gold–palladium. The sample was observed via a secondary electron detector (SE) at an electron beam energy of 15 keV.

Initial PET and MP particles were analyzed via Fourier transform infrared spectroscopy (FTIR) on a Bruker Vector 22 FTIR spectrometer (Bruker, Karlsruhe, Germany) with an ATR PIKE MIRacle accessory with a diamond crystal [34]. The obtained spectra were converted into transmission mode via OPUS 6.0 software.

### 4.3. Animals and MP Administration

Male Wistar rats and senescence-accelerated OXYS rats were obtained from the Center for Genetic Resources of Laboratory Animals at the ICG SB RAS (RFMEFI61914X0005 and RFMEFI61914X0010). At the age of 4 weeks, the pups were weaned, housed in groups of five animals per cage (57 × 36 × 20 cm^3^), and kept under standard laboratory conditions (22 ± 2 °C, on a 12 h light/12 h dark cycle, with lights on at 9 a.m.). The animals had access to standard rodent feed (PK-120-1; Laboratorsnab, Ltd., Moscow, Russia) and water ad libitum.

To evaluate the impact of oral MP administration, animals from the experimental groups of Wistar and OXYS rats from the age of 1.5–3.5 months (the period of active manifestation of signs of accelerated senescence in OXYS rats) received MP particles (polyethylene terephthalate) with food, 2–6 microns in size, at a dose of 10 or 100 mg/kg of MPs in bread balls once per day 5 times a week; control animals received only balls (n = 15 for each group). In this case, we took into account the weight gain that we monitored during the course of the experiment.

### 4.4. Ophthalmoscopic Examinations

Ophthalmoscopic examinations of OXYS and Wistar rats were carried out via a Betta direct ophthalmoscope (HEINE Optotechnik, Gilching, Germany) equipped with a slit lamp after dilatation with 1% tropicamide. All animal procedures were in compliance with the Association for Research in Vision and Ophthalmology statement for the Use of Animals in Ophthalmic and Vision Research as well as the European Communities Council Directive No. 86/609/EES. The stages of cataracts and retinopathy were assessed according to the Age Related Eye Disease Study (AREDS) grade protocol (http://eyephoto.ophth.wisc.edu, accessed on 1 November 2005).

Behavioral testing began 2 days after ophthalmoscopy examination.

### 4.5. Barnes Maze

Two months after supplementation with MPs, the rats were habituated and tested in the Barnes maze. The rats used for Barnes maze analysis were Wistar (n = 30; 10 control, 10 with a dose of 10 mg/kg, and 10 with a dose of 100 mg/kg) and OXYS (n = 30; 10 control, 10 with a dose of 10 mg/kg, and 10 with a dose of 100 mg/kg) rats.

The Barnes maze was used to test spatial memory. Testing was carried out in a setup manufactured by RPC OpenScience, Russia, model TS1101-R (field diameter 122 cm; 18 holes are located around the perimeter). Video tracking and registration of behavioral parameters were carried out via the program EthoVision XT 15 (Noldus, Wageningen, The Netherlands). The protocol consists of habituation, acquisition training (learning), and probing [35].

Habituation and learning trials. During habituation day 1 and all subsequent days (once a day for 2–5 days), each rat was placed in the center of the table, and the animal was allowed to explore the maze. Each rat was allowed 180 s to locate and enter the escape box during each trial. If the rat was unable to locate the escape hole after 180 s, it was gently guided to the correct hole location and allowed to enter the escape tunnel. Once the rat entered the escape tunnel (either guided or on its own), it remained in the tunnel for 30 s before returning to its home cage.

Probe trial. A probe trial was performed 48 h after the last learning day. During this single trial, on day 7, the rats were placed on the table as described for the learning trials. After entering the escape box, the rat was removed and placed back into its holding cage.

Noncognitive behavior. The following parameters were calculated: (1) primary latency (s), (2) distance moved (in cm), (3) speed (cm/s), (4) number of holes, and (5) number of head dips from the table on day 1 as parameters of exploratory activity [36].

Search strategies and development of the cognitive index. To analyze Barnes maze performance, each trial was scored using a modified scale and paths of the possible strategies that a rat can choose to escape [37,38]. Briefly, the scores depended on the hippocampal strategies, which are direct, corrected, focused, and long correction. Nonhippocampal strategies included serial search, random search, and failure. The cognitive index was created by summing the scores for all 4 days. The data were subsequently analyzed in terms of the percentage of trials with the possible strategies by summing the number of learning trials (2–5 days) and separately summing the number of probe trials. For the probe trial, the percentage of time spent in the target quadrant was used to quantify the animal’s ability to insist on the location of the escape hole. The quadrants contain 4–5 possible locations for escape, and the escape hole is in the middle of the target quadrant.

### 4.6. Determination of Hematological and Biochemical Blood Parameters

All groups of rats were euthanized via CO_2_ asphyxiation and decapitation. After that, the organs (liver, heart, kidneys, testes, and brain) were carefully removed and weighed to estimate their mass relative to body weight. Blood samples were taken for immediate analysis of hematological parameters. The remaining blood was centrifuged; the serum was frozen at −20 degrees and then used to determine biochemical parameters. Blood samples were taken for determination of hematological and lipid profiles as well as liver and kidney function parameters.

### 4.7. Hematological Assays

Complete blood counts were performed from peripheral blood samples collected in a volume of 20 µL and added to tubes containing a standardized amount of diluent. After 15 min, the samples were analyzed for the following hematological parameters: white blood cell (WBC) count, including absolute counts of lymphocytes, monocytes, and granulocytes; red blood cell (RBC) and platelet (PLT) counts; hemoglobin (HGB); hematocrit (HCT); mean corpuscular volume (MCV); mean corpuscular hemoglobin (MCH); mean corpuscular hemoglobin concentration (MCHC); red cell distribution width (RDW); and the platelet distribution width (PDW), mean platelet volume (MPV), and platelet count (PCT) using an automated MINDRAY BC-2800 Vet Hematology Analyser (Shenzhen Mindray Animal Medical Technology Co., Ltd., Shenzhen, China).

### 4.8. Biochemical Assays

The blood was centrifuged at 3000× *g* for 15 min within 4 h after collection to obtain the serum, which was immediately stored in Eppendorf tubes at −20 °C until analysis. Serum samples were analyzed for the following parameters: aspartate aminotransferase (AST), alanine aminotransferase (ALT), total cholesterol (TC), triglyceride (TG), total protein (TP), glucose (GLU), alkaline phosphatase (ALP), high-density lipoprotein (HDL), low-density lipoprotein (LDL), creatine kinase, and bilirubin. The quantification of the blood parameters was performed using standard diagnostic kits (Vector-Best, Novosibirsk, Russia) and a Multiscan Ascent photometer (Thermo Labsystems, Helsinki, Finland) according to the manufacturer’s instructions.

### 4.9. Statistical Analysis

The data were subjected to three-way analysis of variance (ANOVA) via Statistica 10.0 software (StatSoft, Tulsa, OK, USA). The genotype, supplementation, and strategy were chosen as independent variables. The Newman–Keuls post hoc test was applied to significant main effects and interactions to assess the differences between some sets of means. The data are presented as the mean ± standard error of the mean (SEM). The differences were considered statistically significant at *p* < 0.05.

## 5. Conclusions

In conclusion, we have shown that, in the absence of changes in standard clinical indicators that definitely indicate health problems, chronic MP intake negative affected the behavior and cognitive functions of both healthy Wistar rats and OXYS rats and contributed to the progression of accelerated aging manifestations in the latter. Additionally, a high dose of MPs contributed to cataract and AMD progression in OXYS rats. Our results suggest that MPs may increase the rate of aging and, in the long term, lifespan.

## Data Availability

The original contributions presented in this study are included in this article/Supplementary Material; further inquiries can be directed to the corresponding authors.

## Supplementary Materials

The following supporting information can be downloaded at: https://www.mdpi.com/article/10.3390/molecules29235776/s1, Table S1: Hematologic blood parameters of Wistar and OXYS rats after exposure to MPs at a dose of 10 or 100 mg/kg. Values were expressed as mean ± SEM; Table S2: Blood biochemical parameters of Wistar and OXYS rats after exposure to MPs at a dose of 10 or 100 mg/kg. Values were expressed as mean ± SEM.
