# Supplementary material for: Invisible but Insidious Effects of Microplastics
Source: Molecules. 2024 Dec 6;29(23):5776. doi: 10.3390/molecules29235776 (PMC11643646; doi:10.3390/molecules29235776)
Supplement: Supplementary file 1 [file molecules-29-05776-s001.zip › molecules-3301833-supplementary.pdf]

molecules MDPI

Table S1. Hematologic blood parameters of Wistar and OXYS rats after exposure MP at a dose of 10 or 100 mg/kg. Values were expressed as mean ± SEM.

	Wistar			OXYS		
Dose of MP	0	10 mg/kg	100 mg/kg	0	10 mg/kg	100 mg/kg
HGB (g/L)	122 ± 16.0	136 ± 10.0 *	129 ± 16.0	136 ± 13.0	126 ± 27.0	124 ± 23.0
RBC (10^{12}/L)	7.50 ± 0.99	8.14 ± 0.46	7.75 ± 0.90	8.47 ± 0.74	7.87 ± 1.64	7.69 ± 1.30
HCT (%)	123 ± 16.8	136 ± 10.0	129 ± 16.1	136 ± 12.9	131 ± 15.0	124 ± 23.1
MCHC (g/L)	325 ± 5.1	326 ± 3.6	326 ± 5.3	325 ± 5.3	322 ± 4.3 #	321 ± 6.3 #
MCV (fL)	51.1 ± 2.39	51.3 ± 1.48	51.1 ± 1.52	49.4 ± 0.70 #	49.5 ± 1.47 #	50.0 ± 0.46
MCH (pg)	16.3 ± 0.49	16.7 ± 0.60	16.6 ± 0.56	16.0 ± 0.29	15.9 ± 0.48	16.0 ± 0.40
WBS (10^9/L)	8.65 ± 3.45	8.74 ± 1.99	8.21 ± 1.91	6.79 ± 1.90	6.82 ± 2.24	6.05 ± 1.91
Lymphocytes (%)	76.9 ± 4.54	76.3 ± 6.76	77.9 ± 3.62	73.2 ± 5.25	72.1 ± 3.24	70.5 ± 4.65
Granulocytes (%)	19.0 ± 4.23	19.0 ± 5.86	17.9 ± 3.07	22.9 ± 4.93 #	23.9 ± 3.12	25.3 ± 4.50
Monocites (%)	4.1 ± 0.6	4.7 ± 1.3	4.2 ± 0.9	3.9 ± 0.7	4.1 ± 0.4	4.2 ± 0.5
PLT (10^9/L)	736 ± 290	742 ± 204	668 ± 190	538 ± 140 #	530 ± 177	464 ± 192
MPV (fL)	5.6 ± 0.8	5.3 ± 0.2	5.3 ± 0.1	6.1 ± 0.2	6.1 ± 0.2	6.1 ± 0.1

* $p < 0.05$ for an effect of MP exposure; # $p < 0.05$ for differences between OXYS and control Wistar rats. HCT: hematocrit; HGB: hemoglobin; MCHC: mean corpuscular hemoglobin concentration MCV: mean corpuscular volume; MPV: mean platelet volume; PLT: platelet; RBC: red blood cell; WBC: white blood cell.

Table S2. Blood biochemical parameters of Wistar and OXYS rats after exposure MP at a dose of 10 or 100 mg/kg. Values were expressed as mean ± SEM.

	Wistar			OXYS		
Dose of MP	0	10 mg/kg	100 mg/kg	0	10 mg/kg	100 mg/kg
Cholesterol (mmol/L)	3.30 ± 0.09	3.25 ± 0.06	3.24 ± 0.03	3.29 ± 0.04	3.27 ± 0.04	3.28 ± 0.07
TG (mmol/L)	1.23 ± 0.04	1.20 ± 0.04	1.22 ± 0.04	1.26 ± 0.11	1.30 ± 0.08	1.28 ± 0.05
TP (g/dL)	8.30 ± 0.20	8.47 ± 0.18	8.29 ± 0.20	8.38 ± 0.32	8.64 ± 0.48	8.52 ± 0.54
Glucose (mmol/L)	6.72 ± 1.51	6.01 ± 0.50	6.18 ± 0.43	6.53 ± 0.49	6.36 ± 0.38	6.47 ± 0.29
LDL (mmol/L)	1.31 ± 0.10	1.38 ± 0.18	1.20 ± 0.09 *	0.98 ± 0.06 #	1.08 ± 0.10 *	1.00 ± 0.13
HDL (mmol/L)	0.78 ± 0.04	0.78 ± 0.02	0.76 ± 0.02	0.80 ± 0.07 #	0.79 ± 0.04	0.80 ± 0.05
Bilirubin (μmol/L)	27.1 ± 3.90	34.3 ± 3.50 *	22.36 ± 5.9	24.1 ± 6.2	33.6 ± 7.0 *	32.3 ± 13.4
ALT (U/L)	26.03 ± 4.96	22.31 ± 3.53	25.51 ± 2.67	22.49 ± 5.07	19.89 ± 4.12	26.98 ± 6.93
AST (U/L)	54.54 ± 10.08	55.45 ± 9.91	54.54 ± 6.33	59.61 ± 10.59	61.44 ± 8.50	59.89 ± 8.32
CK (U/L)	334 ± 86.7	379 ± 43.9	302 ± 94.5	308 ± 55.7	384 ± 64.1	338 ± 91.0
ALP (U/L)	137 ± 12.3	139 ± 34.2	147 ± 39.8	112 ± 18.0	104 ± 13.6	121 ± 26.4

* $p < 0.05$ for an effect of MP exposure; # $p < 0.05$ for differences between OXYS and control Wistar rats. ALT: alanine aminotransferase; AST: aspartate aminotransferase; ALP: alkaline phosphatase; CK: creatine kinase; LDL: low-density lipoprotein; HDL: high-density lipoproteins; TG: triglyceride; TP: total protein.
